# Supplementary material for: Bull Shark (Carcharhinus leucas) Occurrence along Beaches of South-Eastern Australia: Understanding Where, When and Why
Source: Biology (Basel). 2023 Aug 31;12(9):1189. doi: 10.3390/biology12091189 (PMC10526001; doi:10.3390/biology12091189)
Supplement: Supplementary file 1 [file biology-12-01189-s001.zip › biology-2520736-supplementary.pdf]

Table S1. Details of tag deployments for 111 bull sharks tagged and detected along the New South Wales coast between 2010 and 2022. Date format is day/month/year. Including the number of: (i) shark listening stations each individual was detected on and (ii) number of IMOS stations each individual was detected on after the last detection on the shark listening station.

ID	Date tagged	Latitude tagged	Type of habitat	Sex	Total length (cm)	Tag placement	Date of first detection	Date of last detection	Size at first detection (TL, cm)	Total number of detections	No. of shark listening stations visited	No. of IMOS stations visited
1466-1467	21/01/2010	-33.846	Coastal	Male	257	Internal	30/12/2017	28/04/2018	257	7	2	0
1452-1453	5/11/2010	-29.385	Estuarine	Female	140	Internal	19/07/2017	30/05/2018	190	68	1	0
1438-1439	7/11/2010	-29.391	Estuarine	Male	128	Internal	7/02/2018	8/01/2019	190	247	5	0
64936	7/11/2010	-29.385	Estuarine	Male	101	Internal	18/07/2017	20/11/2019	190	79	8	0
1442-1443	6/12/2010	-29.422	Estuarine	Male	155	Internal	14/01/2018	31/05/2018	190	16	5	0
64931	6/12/2010	-29.303	Estuarine	Female	80	Internal	26/04/2019	16/12/2020	183	102	7	1
1446-1447	7/12/2010	-29.431	Estuarine	Female	102	Internal	28/12/2017	31/12/2017	302	3	2	0
64927	10/12/2010	-29.422	Estuarine	Female	145	Internal	25/01/2018	14/03/2020	190	13	2	1
1458-1459	12/12/2010	-29.422	Estuarine	Female	320	Internal	12/11/2018	6/03/2020	320	39	4	0
64317	1/02/2011	-33.842	Coastal	Male	260	Internal	6/01/2018	16/02/2019	260	21	3	6
64321	4/02/2011	-33.842	Coastal	Female	264	Internal	17/02/2020	14/03/2020	264	5	2	0
31930	2/03/2011	-29.416	Estuarine	Female	148	Internal	7/02/2018	25/03/2019	190	28	4	33
31938	2/03/2011	-29.426	Estuarine	Female	190	Internal	19/10/2017	6/02/2020	190	120	11	0
14685	21/01/2016	-33.864	Coastal	Male	223	Internal	3/01/2018	28/12/2022	223	16	3	0
20462	3/02/2016	-28.870	Estuarine	Male	176	Internal	13/08/2017	2/05/2020	190	66	6	1
20464	3/02/2016	-28.878	Estuarine	Female	152	Internal	13/07/2017	4/11/2022	170	908	9	0
20470	4/02/2016	-28.877	Estuarine	Male	187	Internal	7/12/2017	16/03/2021	187	204	5	9
20461	1/03/2016	-33.847	Coastal	Male	273	Internal	26/02/2019	13/12/2019	273	15	5	26
20463	1/03/2016	-33.845	Coastal	Male	249	Internal	9/12/2017	2/05/2020	249	19	5	22
20467	3/03/2016	-33.854	Coastal	Male	280	Internal	27/12/2017	16/03/2021	280	15	4	16
20466	7/03/2016	-33.841	Coastal	Male	274	Internal	17/04/2019	17/04/2019	274	4	1	21
18829	14/12/2016	-31.878	Estuarine	Female	168	Internal	24/07/2017	18/07/2021	175	628	13	14
18767	19/12/2016	-30.458	Coastal	Male	160	Internal	14/07/2017	6/05/2021	168	1736	11	9
18759	20/12/2016	-30.446	Coastal	Female	290	Internal	9/12/2018	30/12/2022	290	45	4	1
29678	13/02/2017	-33.845	Coastal	Female	231	Internal	28/10/2017	24/04/2018	231	8	3	0
29679	13/02/2017	-33.841	Coastal	Male	300	Internal	30/12/2017	30/12/2017	300	3	1	25

18831	16/02/2017	-30.490	Estuarine	Female	173	Internal	26/02/2018	15/05/2021	184	763	7	17
29680	21/02/2017	-33.855	Coastal	Male	220	Internal	2/01/2018	1/03/2021	220	46	12	17
18753	23/02/2017	-28.877	Estuarine	Male	158	Internal	11/02/2018	27/05/2020	172	550	8	0
18827	23/02/2017	-28.875	Estuarine	Male	162	Internal	24/07/2017	10/08/2019	168	158	4	0
18755	24/02/2017	-28.875	Estuarine	Male	151	Internal	21/07/2017	12/07/2021	157	481	6	12
18757	24/02/2017	-28.878	Estuarine	Male	153	Internal	13/07/2017	13/04/2021	159	547	8	12
29683	2/03/2017	-33.848	Coastal	Male	278	Internal	8/02/2019	15/03/2019	278	4	2	9
12545	15/11/2017	-31.877	Estuarine	Female	291	External	27/11/2017	2/02/2018	291	305	3	0
14230	20/11/2017	-30.447	Coastal	Female	155	Internal	6/04/2020	6/07/2020	182	76	4	10
18784	20/11/2017	-30.452	Coastal	Female	158	Internal	17/06/2019	14/06/2021	176	324	8	10
14270	23/11/2017	-30.490	Coastal	Female	178	Internal	17/10/2018	31/03/2020	187	125	7	10
16429	23/11/2017	-28.882	Coastal	Female	290	External	24/11/2017	30/11/2017	290	47	3	0
14263	29/11/2017	-28.879	Estuarine	Female	277	Internal	2/12/2017	5/12/2022	277	155	12	3
14269	29/11/2017	-28.878	Estuarine	Female	158	Internal	26/03/2018	9/06/2021	162	561	6	5
18785	29/11/2017	-28.877	Estuarine	Female	303	Internal	1/12/2019	19/12/2019	303	20	2	0
12547	7/01/2018	-31.877	Estuarine	Female	286.5	External	10/01/2018	10/01/2018	287	12	1	0
29697	11/01/2018	-33.846	Coastal	Male	204	Internal	16/02/2019	2/02/2021	204	7	2	13
18826	18/01/2018	-33.846	Coastal	Male	270	Internal	4/01/2019	19/03/2021	270	11	5	25
20468	18/01/2018	-33.846	Coastal	Female	295	Internal	4/12/2019	4/12/2019	295	1	1	8
20469	22/01/2018	-33.855	Coastal	Female	292	Internal	27/04/2018	2/01/2023	292	68	5	6
12950	23/01/2018	-28.841	Coastal	Female	254	External	27/10/2018	11/03/2021	254	54	2	0
18828	24/01/2018	-33.844	Coastal	Male	270	Internal	3/02/2018	11/03/2021	270	9	3	21
18750	29/01/2018	-33.853	Coastal	Female	265	Internal	11/02/2018	11/02/2018	265	2	1	0
18754	15/02/2018	-33.849	Coastal	Male	285	Internal	7/01/2019	7/03/2021	285	15	5	16
18824	15/02/2018	-33.837	Coastal	Male	292	Internal	7/05/2018	7/05/2018	292	1	1	37
18762	21/02/2018	-33.837	Coastal	Male	255	Internal	16/04/2018	6/01/2021	255	45	6	17
18822	21/02/2018	-33.836	Coastal	Female	292	Internal	28/03/2018	11/03/2019	292	20	7	7
18752	22/02/2018	-33.846	Coastal	Male	249	Internal	7/03/2018	30/12/2022	249	53	11	3
18763	27/02/2018	-33.836	Coastal	Male	270	Internal	26/03/2018	8/04/2021	270	2	2	8
12951	26/04/2018	-28.832	Coastal	Male	193	External	26/04/2018	10/07/2020	193	101	3	1
12279	3/05/2018	-28.882	Coastal	Female	180	External	9/05/2018	18/06/2018	180	190	6	2
12298	6/05/2018	-29.113	Coastal	Female	172	External	12/05/2018	13/08/2018	172	278	6	0
12317	16/06/2018	-29.098	Coastal	Male	181	External	20/06/2018	6/11/2018	181	26	4	0
12548	16/11/2018	-31.873	Estuarine	Male	196	External	26/02/2019	14/11/2019	196	1166	8	0
12549	18/11/2018	-31.873	Estuarine	Female	310	External	25/11/2018	25/11/2018	310	8	2	3
12551	25/12/2018	-31.873	Estuarine	Male	288	External	26/12/2018	13/01/2021	288	58	5	0

11979	29/01/2019	-33.843	Coastal	Male	237	Internal	27/02/2019	2/01/2023	237	52	10	0
18830	29/01/2019	-33.842	Coastal	Male	272	Internal	17/03/2020	9/03/2021	272	28	6	0
11980	30/01/2019	-33.842	Coastal	Male	290	Internal	29/04/2020	12/05/2021	290	18	6	0
11981	30/01/2019	-33.864	Coastal	Male	297	Internal	25/02/2019	26/12/2022	297	49	8	0
11982	12/02/2019	-33.854	Coastal	Male	281	Internal	28/02/2019	13/04/2021	281	41	4	0
11983	19/02/2019	-33.848	Coastal	Male	252	Internal	11/05/2019	8/12/2022	252	101	11	0
11984	19/02/2019	-33.841	Coastal	Male	287	Internal	31/03/2020	13/03/2021	287	47	1	0
11985	6/03/2019	-33.841	Coastal	Male	285	Internal	6/02/2020	30/04/2021	285	120	4	0
11991	19/03/2019	-33.853	Coastal	Male	251	Internal	11/02/2021	22/03/2021	251	24	1	0
11986	21/03/2019	-33.864	Coastal	Female	255	Internal	6/04/2019	11/12/2022	255	77	16	0
12552	22/03/2019	-32.330	Coastal	Female	280	External	8/11/2019	10/11/2019	280	21	5	0
11987	27/03/2019	-33.864	Coastal	Female	207	Internal	16/04/2019	5/04/2021	207	74	12	0
12555	6/04/2019	-32.330	Coastal	Male	240	External	4/05/2019	4/05/2019	240	5	1	0
12556	22/11/2019	-31.871	Estuarine	Female	188	External	18/02/2020	18/02/2020	188	3	1	0
8132	10/01/2020	-31.877	Estuarine	Male	226	External	21/01/2020	29/03/2021	226	77	6	0
5838	19/02/2020	-29.105	Coastal	Female	190	External	20/02/2020	20/02/2020	190	5	1	8
12020	19/03/2020	-33.854	Coastal	Male	207	Internal	8/12/2020	2/03/2021	207	28	7	0
11988	20/01/2021	-33.846	Coastal	Female	241	Internal	23/02/2021	1/01/2023	241	17	3	0
4326-4327	22/01/2021	-29.113	Coastal	Male	195	External	9/02/2021	8/12/2022	195	48	12	0
11990	1/02/2021	-33.841	Coastal	Male	286	Internal	10/03/2021	30/12/2022	286	4	3	0
11993	1/02/2021	-33.846	Coastal	Female	309	Internal	23/02/2021	2/03/2021	309	8	2	0
12017	1/02/2021	-33.854	Coastal	Female	250	Internal	8/02/2021	19/11/2022	250	36	8	0
11989	4/02/2021	-33.844	Coastal	Female	264	Internal	10/03/2021	16/11/2022	264	17	5	0
12019	4/02/2021	-33.840	Coastal	Male	255	Internal	11/02/2021	21/04/2021	255	21	2	0
11997	18/02/2021	-33.846	Coastal	Female	230	Internal	11/03/2021	31/12/2022	230	35	11	0
4302-4303	25/02/2021	-28.839	Coastal	Female	177	External	25/03/2021	27/04/2021	178	277	1	0
4304-4305	26/02/2021	-29.113	Coastal	Female	185	External	5/03/2021	28/12/2022	185	1446	6	0
4322-4323	28/03/2021	-28.884	Coastal	Female	175	External	5/04/2021	26/04/2021	175	10	2	0
4344-4345	17/04/2021	-28.868	Coastal	Female	195	External	17/04/2021	18/04/2021	195	4	1	0
4182-4183	6/12/2021	-29.090	Coastal	Female	283	External	12/12/2022	30/12/2022	283	25	4	0
4174-4175	12/01/2022	-29.090	Coastal	Female	185	External	19/11/2022	26/12/2022	185	65	1	0
3802-3803	15/01/2022	-29.435	Coastal	Male	182	External	6/12/2022	28/12/2022	182	62	3	0
3934-3935	27/01/2022	-28.848	Coastal	Male	182	External	7/01/2023	7/01/2023	182	5	2	0
3914-3915	10/02/2022	-28.832	Coastal	Male	188	External	21/11/2022	23/11/2022	188	41	2	0
3962-3963	15/02/2022	-28.873	Coastal	Male	186	External	15/12/2022	22/12/2022	186	28	5	0
3992-3993	8/03/2022	-28.309	Coastal	Male	203	External	10/12/2022	10/12/2022	203	1	1	0

13230-13231	23/03/2022	-28.881	Coastal	Male	173	External	9/11/2022	2/12/2022	181	19	3	0
3920-3921	23/03/2022	-30.273	Coastal	Female	178	External	15/12/2022	7/01/2023	185	64	5	0
13835-13836	11/04/2022	-29.098	Coastal	Female	242	External	10/12/2022	21/12/2022	242	24	1	0
13224-13225	29/04/2022	-28.824	Coastal	Female	204	External	4/01/2023	4/01/2023	204	6	1	0
13226-13227	22/05/2022	-30.353	Coastal	Male	196	External	10/12/2022	10/12/2022	196	13	1	0
3770-3771	16/06/2022	-29.476	Coastal	Female	188	External	4/11/2022	10/11/2022	188	26	2	0
61036	14/11/2022	-30.880	Coastal	Female	293	External	17/11/2022	24/12/2022	293	77	9	0
61039	14/11/2022	-30.880	Coastal	Female	302	External	17/11/2022	27/11/2022	302	54	1	0
61040	19/11/2022	-30.755	Coastal	Female	302	External	23/11/2022	16/12/2022	302	54	6	0
4024-4025	25/11/2022	-30.273	Coastal	Female	165	External	26/11/2022	26/11/2022	165	7	1	0
4006-4007	5/12/2022	-29.416	Coastal	Female	183	External	6/12/2022	6/12/2022	183	4	1	0
11969-11970	6/12/2022	-29.113	Coastal	Male	213	External	18/12/2022	24/12/2022	213	14	1	0
11300-11301	14/12/2022	-30.284	Coastal	Female	197	External	22/12/2022	9/01/2023	197	10	1	0

Table S2. Minimum, maximum, mean and standard error of the number of days those individuals were detected along the coast of NSW.

Group	Min	Max	Mean	Standard Error
Juveniles (80-174 cm)	1	50	21.9	5.3
Large males (176-312 cm)	1	103	13.7	2.6
Large females (175-322 cm)	1	117	15.9	3.2

Table S3. Variable selection procedure of generalized additive mixed models of large bull shark occurrence along the coast of NSW, comprising the candidate explanatory environmental variables daily rainfall (Rain), swell height (Height), tidal height (Tide), water temperature (Temp) and moon phase (Moon). Included are the respective Akaike Information Criterion (AIC), delta AIC (ΔAIC) and Akaike weights (AIC_w). (*) Variables discarded due to lack of statistical significance.

Model	AIC	ΔAIC	AIC_w
Null	40531.28	3378.04	< 0.0001
Rain	40524.96	3371.72	< 0.0001
Height	40123.11	2969.87	< 0.0001
Tide*			
Temp	37279.51	126.27	< 0.0001
Moon	40523.93	3370.69	< 0.0001
Temp + Rain	37188.13	34.89	< 0.0001
Temp + Height	37261.72	108.48	< 0.0001
Temp + Moon*			
**Temp + Rain + Height**	**37153.24**	**0**	**0.9999**

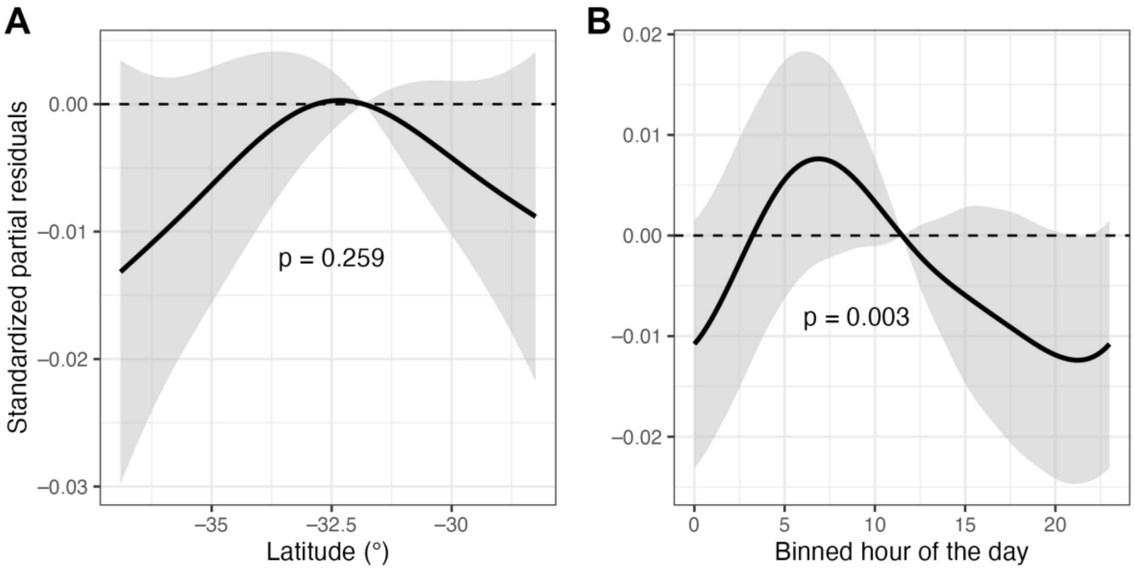

Figure S1: Shark listening station detection probabilities as a function of the variables (A) latitude (non-significant) and (B) hour of the day (significant).

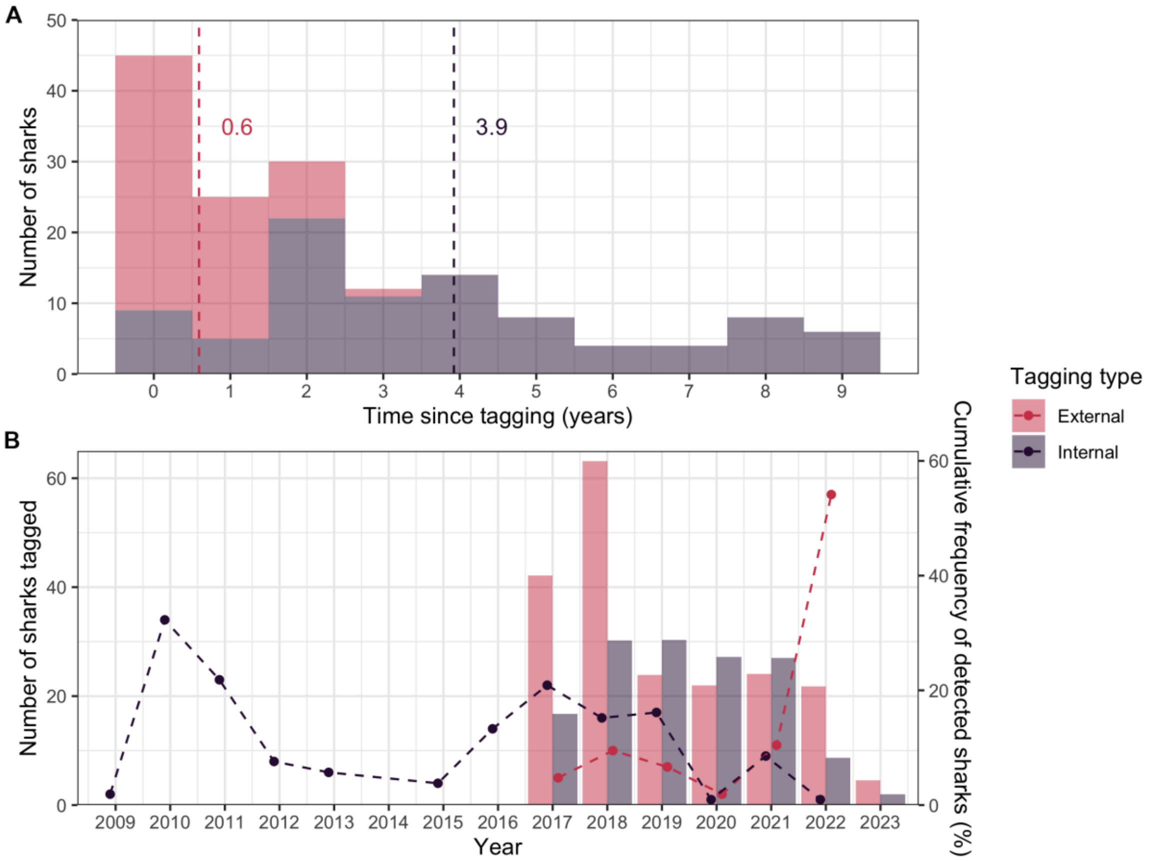

Figure S2: Histograms of acoustic detections (A) as a function of time since tagging and (B) yearly number of sharks tagged (point) together with their corresponding frequencies of redetection (bars), for externally and internally tagged bull sharks.

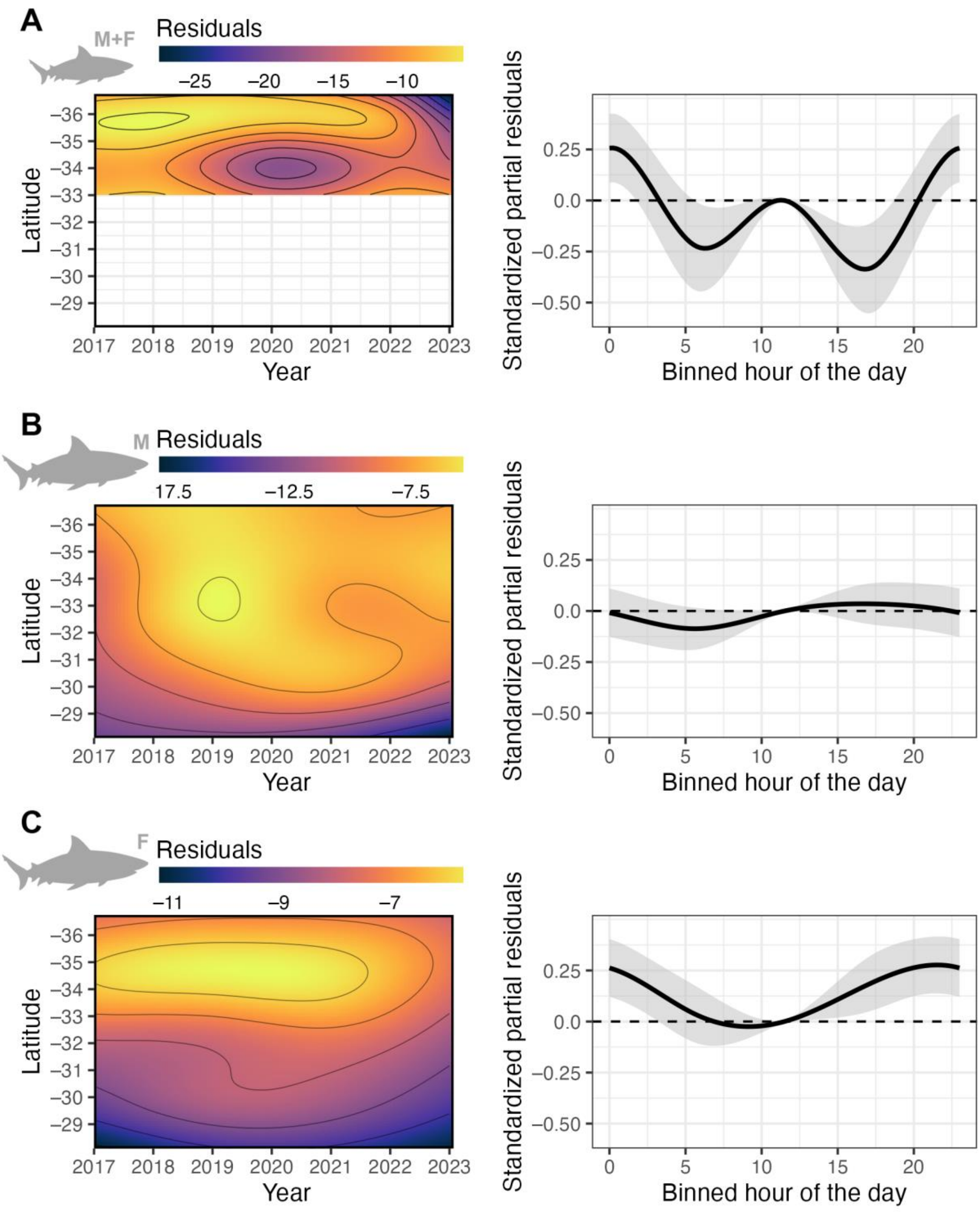

Figure S3: Group-specific spatio-temporal generalized additive models of (A) juvenile, (B) large male and (C) large female bull shark occurrence along the coast of New South Wales as a function of the interacting effects between latitude degree and year (left panel) and binned hour of the day (right panel). The colour scales (left panel) represent the corresponding fitted model residuals. Horizontal dashed lines and shaded areas (right panel) respectively represent the null effects and the 95% confidence intervals. Positive values on the vertical axis indicate an increased probability of occurrence, while negative values indicate an increased probability of absence (right panel).

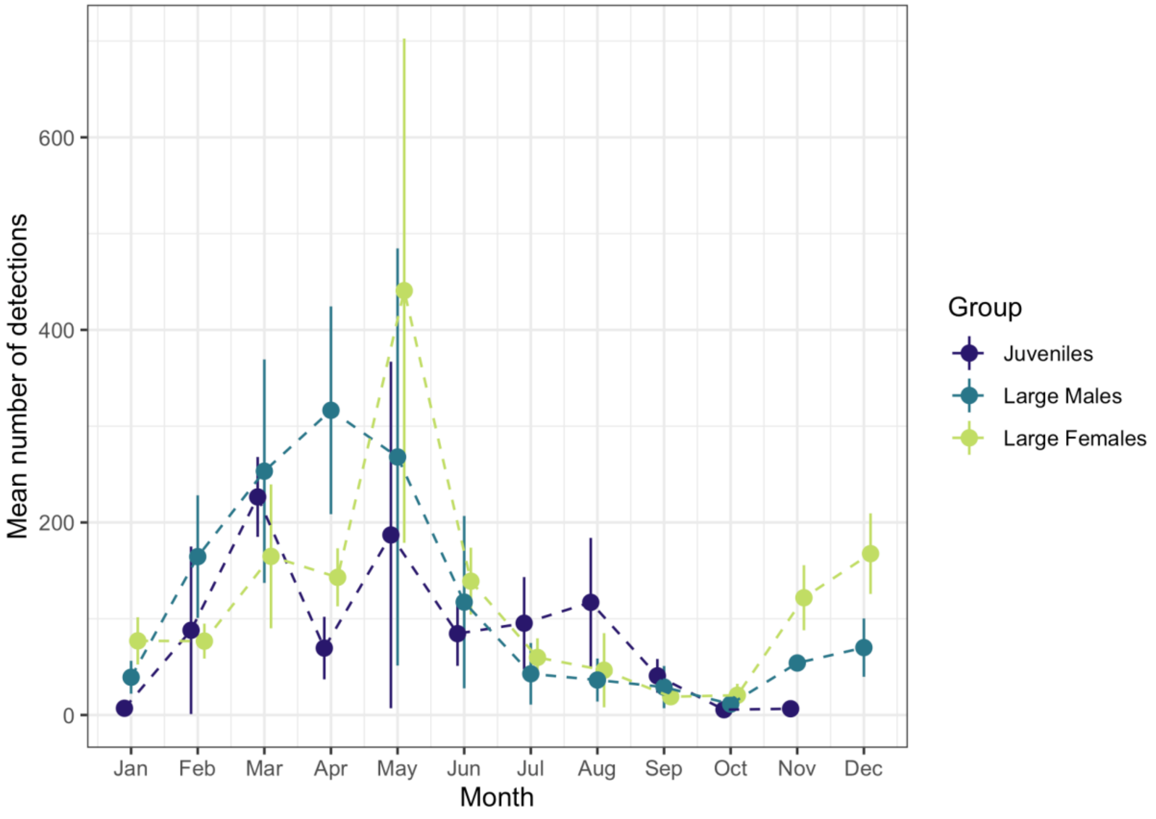

Figure S4: Mean (± SE) monthly occurrence of juveniles (80-174 cm TL), large males (176-312 cm TL) and large female (175-322 cm TL) bull sharks along the coast of New South Wales.

Data sources and dates of download:

Distance to river
https://www.environment.gov.au/fed/catalog/search/resource/details.page?uuid=a8bd8d06-813b-4420-908b-a79c065cf533; downloaded on 26 April 2023.

Manly Hydraulics

https://mhl.nsw.gov.au/; downloaded on 26 April 2023.

Bureau of Meteorology

http://www.bom.gov.au/climate/data/; downloaded on 26 April 2023.
